# Rorschach Evaluation of Personality and Emotional Characteristics in Adolescents With Migraine Versus Epilepsy and Controls

**DOI:** 10.3389/fneur.2018.00160

**Published:** 2018-03-20

**Authors:** Laura Balottin, Stefania Mannarini, Daniela Candeloro, Alda Mita, Matteo Chiappedi, Umberto Balottin

**Affiliations:** ^1^Interdepartmental Center for Family Research, Department of Philosophy, Sociology, Education, and Applied Psychology, Section of Applied Psychology, University of Padova, Padova, Italy; ^2^Child Neuropsychiatry Unit, Department of Brain and Behavioral Sciences, University of Pavia, Pavia, Italy; ^3^Child Neuropsychiatry Unit, IRCCS Mondino Foundation, Pavia, Italy

**Keywords:** adolescence, headache, affective regulation, migraine, personality traits, psychopathology, psychiatric symptoms, Rorschach test Exner comprehensive system

## Abstract

The literature examining primary headache, including migraine, in adolescents, has pointed out the key role played by a wide range of psychiatric disorders in reducing the patients’ quality of life. Moreover, pioneering studies showed that preexisting personality characteristics, specific emotion regulation styles and psychological-psychiatric difficulties are likely to increase the risk of the onset, maintenance, and outcome of headache. Still personality issues in migraine have been poorly studied, in particular in children and adolescents. This study aims, therefore, to investigate the specific characteristics of personality, and in particular emotion regulation and coping strategies, in adolescent with migraine, comparing them with age-matched patients with idiopathic epilepsy and healthy adolescents. 52 adolescents (age: 11–17) were assessed using a multi-method test battery, which included a self-report questionnaire (the youth self-report), a proxy-report (child behavior checklist) along with a projective personality test, the Rorschach Test, administered and scored according to the Exner comprehensive system. The results showed specific personality characteristics in adolescents with migraine, revealing a marked difficulty in modulating and regulating affections through thoughts and reflections, resorting instead to impulsive acts and maladaptive coping strategies, thus revealing a vague and immature perception of reality. Differently from adolescents belonging to the general population, but similarly to patients with epilepsy, adolescents with migraine perceive a high situational stress, probably related to the condition of suffering from chronic disease. They have, therefore, a lower self-consideration and self-esteem along with a poorer insight regarding themselves as well as the relations with others. In line with previous findings, these preliminary results suggest the need for further research on ample samples, using also standardized projective test in order to better understand the pathogenesis of psychological difficulties in patients with migraine. As a clinical implication, the results seem to indicate that providing a psychological integrated approach can play a pivotal role in the assessment and treatment of adolescent with migraine, in order to improve the outcome and the quality of life of the young patients.

## Introduction

Personality and emotional characteristics in adolescents with migraine began to be more in-depth studied in recent years: literature showed an increased interest in emotional aspects, such as anxiety and depression, and in their value for the clinical approach, therapy, and comprehension of the adolescent migraine’s key characteristics (1–5).

The majority of the authors currently consider migraine as a complex neurological disorder of higher mental functions and pain control mechanism, not linked to structural lesions: in particular strong pathogenic mechanisms have been recognized in genetic predisposition, cortical hyperexcitability, habituation deficits, and cognitive dysfunctions, instability of the autonomic nervous system and of the neurons’ energy supply or in other cases medication overuse (6, 7). Different authors consider the disorder as a dysfunction of the neuromodulatory structures in the brain, such as the locus coeruleus or the periaqueductal gray matter (8). Quite well-known are the biological mechanisms that are related to migraine in adulthood as well as in adolescence and the hypothesis of migraine as a disorder caused by a neurovascular dysfunction is nowadays the most accepted. The genetic bases, biological mechanisms, cerebral blood flow variations during attacks, electrical alterations occurring in parallel and biochemical modifications seem to have been deeply studied, leading to certain conclusions, which seem almost inviolable; on the other hand, however, the definition and comprehension of the environmental factors’ value, and in particular of the psychological and psychiatric ones, seems to be still far more complex and uncertain (9).

It is worth noticing that paradoxically adolescent migraine is much more sensitive to “psychological” than to pharmacological interventions. Relevant meta-analyses (10–13) and recent high impact researches (14) showed a lack of efficacy of the candidate drugs for the prevention of migraine attacks in adolescence and, on the other hand, a good effectiveness of the “psychological” techniques. Placebo indeed was showed to have a surprisingly high therapeutic value: a 50% reduction (at least) of the days with headache was in fact achieved in about 60% of patients (14) and in 58% of the placebo also produced a positive response when used as a pain-reliever for migraine attacks (15).

In conclusion, while important evidence about the biological basis of adolescent migraine was established, at the same time a lack of sensitivity of the pathology to the currently known pharmacological therapies was shown. The relationships between migraine and emotional states affect and personality characteristics appear instead very much complex and difficult to investigate and definitively define; so far in fact different and contradictory results were found. Population studies, such as those by Antilla, Aromaa, and Sillampaa (16), detected significantly more marked total, internalizing, and somatic symptoms, as well as family and social problems, in children with migraine compared to controls; nevertheless they also found that “only a minority of children with migraine or tension-type headache have high levels of psychiatric symptoms.” Similarly, a recent systematic review (17) showed a relationship between migraine and somatic complaints and internalizing disorders, but interpreted the correlation as “a consequence of the nature of the disease rather than a sign of psychological dysfunctioning.”

However, several findings might also suggest a different hypothesis on the pathogenic role of personality and psychopathological characteristics in child and adolescent migraine. Well-known authors from different scientific backgrounds, such as Kandel (18) and Marty (19) have in fact always emphasized the constant bidirectional interactions between mind and brain. Controlled clinical studies (20–22) and rigorous meta-analyses (23) demonstrated that children and adolescents with migraine and tension-type headache show a significantly higher prevalence of psychopathological symptoms compared to healthy controls, in particular concerning internalizing disorders, such as anxiety and depression; oppositional defiant disorder and conduct disorders were also demonstrated to be more frequent in these patients (24). Furthermore, a population-based study conducted on 1,135 Finnish children (16) found not only more internalizing and total behavioral problems (28.8%) in children suffering from migraine, but also more marked difficulties in family functioning and social relationships.

Additionally, concerning population-based studies, which offer a high degree of evidence in terms of evidence-based medicine, interesting aspects emerged concerning the timing of the onset of the two disorders, i.e., headache and subsequent psychiatric disorders later in life or *vice versa*, psychiatric disorders and subsequent migraine. According to a cohort study conducted on 17.414 children (25), children with recurrent headache exhibited an increased risk not only for new recurrent attacks of headache and/or migraine, but also for numerous other somatic symptoms (OR 1.75) and psychiatric disorders (OR 1.41) in adulthood. Fearon and Hotopf (25), therefore, concluded that child physical symptoms, such as recurrent headache and migraine, might represent “signs of an underlying psychosocial adversity.” “Migraine should represent a subtype of headache of particular interest for psychiatrists” because of the significant association “between stress, personality traits, psychiatric disorders and migraine” as found by Cahill (26) in a cohort of children born in Dunedin (New Zealand). This large and rigorous longitudinal study (26) reported that 9 years old children and 15 years old adolescents with high levels of stress and anxiety exhibited a 2–3 times increased risk of later developing migraine or migraine along with tension-type headache.

Even more interestingly from the point of view of physiopathology, the presence of psychiatric disorders in the parents’ correlates with the presence of migraine disorder in children. In a sample of 674, 17 years old girls, drawn from the Minnesota Twin Family Study, a community-based study of adolescents and their families, Marmorstein (27) established that “parental depression, antisocial behavior and drug dependence were associated with offspring migraine.” Indeed observing with specific expertise children with migraine and their families may suggest the existence of deep family conflicts, often along with adverse life events, such as parents’ separations or divorce, and excessive parental expectations concerning their child. Specific children’s personality characteristics also appear frequently related to this milieu: children often seem hyper-responsible or taking on a protective role toward the parents; moreover, they exhibit rigid and normative personality and a very severe attitude toward themselves (28). A reduced emotion regulation (ability to get in contact and psychologically handle feelings and emotions) and low empathic skills were also highlighted in patients with migraine (29, 30), despite these latter characteristics seemed to be ignored by the patients themselves, due to their inclination to deny any difficulty, tension, and in particular hostile feelings and negative emotions (28, 31, 32).

Although, to the current state of knowledge, psychological factors seem to play an important role in headaches, the correlation remains unclear and raises a wide-ranging debate (33). Despite the good number of studies predominantly concerning the comorbidity of headache with psychiatric symptoms and their course over time, the personality characteristics and functioning of adolescents with migraine are still unclear. Searching with the keywords “Migraine” AND “Adolescence” (-Adolescent) AND “Personality,” PubMed finds only one study (20). Moreover, this as well as most of the above-mentioned studies, used only standardized self-administered scales and questionnaires, which exclusively explore the patients’ or parents’ point of view through multiple-choice questions that can be eluded by defense mechanisms, such as normalization and denial. Such diagnostic tools are, however, meant to measure dimensions of personality structure, which often cannot be readily accessible to the patient’s consciousness, especially considering the low age of the samples and the potential presence of personality pathologies. Despite their psychometric properties, often validated and supported by plenty of scientific literature, these instruments frequently remain at a superficial diagnostic level, which is a strong limitation for a deep understanding of psychopathology (31, 33). To date, most of the studies evaluating psychopathological characteristics in patients with headaches are based only on the proxy-reported child behavior checklist (CBCL); this, however, leads to a need to enrich the assessment in order to more specifically capture the relationship between migraine and psychopathology (23).

This study, investigating psychological characteristics and psychopathology in a sample of adolescents with migraine, based, therefore, on a multi-method assessment that combines the perspective of the patient (self-report) and of the parents (proxy-report) with a projective test. The application of the Rorschach test administered and scored according to the Rorschach Comprehensive System (CS) guidelines aimed to describe the deep personality functioning and to identify the presence of any specific dysfunctional element in the sample of patients (34). Some previous studies on adolescent migraine have used the Rorschach method, according to the unpublished traditional administration and scoring techniques (35, 36). Such researches provided a wealth of interesting findings on the personality profile of children and adolescents with migraine, but appeared to be also partially contradictory due to the non-standardization of the methods and to the bias in the mean ages of the groups compared. Summarizing the results, the researches agreed in finding a low level of insight and empathy in children with migraine, along with some specific personality difficulties, such as inhibition of affects, hyper-adaptation, and weakness of the self.

Differently from the above-mentioned studies, this research specifically relies on a standardized performance-based personality test, the Rorschach test administered and scored according to the Rorschach CS, which provides implicit personality functioning characteristics and highlights the unconscious aspects of complex cognitive behaviors, emotional and decision-making processes (34). Additionally, the Rorschach test applied according to the CS has been subjected to a sophisticated meta-analysis which demonstrated that 13 of the variables identified by the CS exhibit an excellent validity against externally assessed criteria, such as psychiatric diagnosis and standardized observer ratings (37). To our knowledge, no previous study used the Rorschach test according to the CS to study patients with migraine, neither in child or adolescent age nor in adulthood.

In this study, the sample of adolescents with migraine was compared with adolescents suffering from a different chronic disorder involving the central nervous system, i.e., idiopathic epilepsy, and with healthy adolescents randomly selected by a home pediatrician among his patients without psychological and neurological problems. This method allowed to differentiate migraine from a paroxysmal disorder with exclusively biological pathogenic mechanisms, such as epilepsy, and also to compare adolescents with migraine to a non-patient control population for what concerns personality characteristics, and in particular emotion regulation and coping strategies. The areas of affectivity, self-perception, interpersonal relationships, ideation, and stress control were explored in the three groups of adolescents, basing, in particular on the subsequent hypotheses:

(1) adolescents with migraine may have a specific difficulty in experiencing, modulating, and expressing emotions and affects, according to the previous mentioned studies (23, 29) which found high levels of psychopathology and alexithymia in these patients,
(2) adolescents with migraine, similarly to those with epilepsy and other chronic illness, have to face a high level of negative emotions and significant situational stress compared to healthy adolescents without migraine,
(3) adolescents with migraine may adopt, however, fragile strategies of coping, compared to controls and patients with epilepsy due to their fragilities in managing emotions.

## Materials and Methods

### Participants and Procedure

Patients with migraine or epilepsy were recruited at the Child and Adolescent Neuropsychiatry Unit, National Neurological Institute IRCCS C. Mondino—University of Pavia (Italy), among patients that had been consecutively admitted during the period of the recruitment, which lasted 7 months. For all participants in the study, inclusion criteria were: age ranging from 11 to 17 years, absence of other chronic physical illness excluding migraine or epilepsy, respectively in the two clinical groups. Exclusion criteria were the presence of visual impairment that could interfere with performance at the Rorschach test or with the administration of self-reports.

The healthy control group was recruited among patients of a home pediatrician of the same city during a routine pediatric check-up. Parents were interviewed in order to collect precise information on medical history, i.e., the family’s physiological, recent and remote, pathological medical history, with a particular focus on any symptoms of headache or epilepsy-like symptom. Medical histories of headaches, epilepsy, or different organic chronic physical diseases were considered as exclusion criteria.

All participants were administered self-reported scales, youth self-report (YSR) for the adolescents and CBCL for the parents (38). An examiner who completed an advanced training on Exner’s CS administered the Rorschach test to the adolescents. Patients and their parents adhered on voluntary basis after detailed explanation of the project. Parents and adolescents were informed and expressed their consent to the participation in the study. As far as migraine and epilepsy patients were concerned, the submitted tests fell within the evaluations carried out within the psycho-pathological assessment.

The study involved 21 patients suffering from migraine (9 males and 13 females) aged 11–17 years (mean 14.7 ± 1.8), 20 patients with epilepsy (11 males and 9 females) aged 11–17 years (mean 14.1 ± 2.2), and 11 adolescents of the control group (4 males and 7 females) aged 12–17 years (mean 14.1 ± 1.9). Most of the adolescents (25 out of 52, i.e., the 48%) ranked in a medium-low socioeconomic level in all the three groups. There were in fact no differences in the socioeconomic level measured with SES (39) as well as in the duration of the disorder among the groups: the mean duration of the disease was 36 months for the headache group versus 40 months for the epilepsy one.

For what concerns specifically the group with migraine, according to the second edition of the International Classification of Headache Disorders (40), 16 (76%) out of the 21 patients had a diagnosis of migraine without aura: among these, 9 have a chronic form of migraine with daily attacks, one patient has 4–6 attacks per month, while 6 have fewer than 3 attacks per month. Concerning the form with aura, 5 (24%) patients were diagnosed with migraine with aura and have 2–6 headache attacks per year. Tension-type headache was associated with migraine in 7 (33%) patients and migraine was chronical in 9 (43%) patients. The majority of the migraineurs (13) does not take any prophylactic therapy, while 4 patients take a therapy based on a combination of l-tryptophan and niacin, 2 patients take magnesium, and 2 other palmitoylethanolamide.

Epilepsies were diagnosed, following the ILAE classification (41) as benign epilepsy with centro-temporal spikes in 8 patients, childhood absence epilepsy in 3 cases, reflex epilepsy in 2 cases, late-onset childhood occipital epilepsy, juvenile absence epilepsy, epilepsy with myoclonic absence, epilepsy with myoclonic atonic seizures, epilepsy with generalized tonic–clonic seizures alone, frontal local seizure, and epilepsy of unknown causes in 1 patient each. Of these patients, 16 are considered in symptomatic remission, as they had no seizures for months, 2 patients still have seizures with a frequency of one a year, 1 patients has seizures with a frequency of 3–4 month and 1 presents myoclonic episodes several times a week, which, however, do not disturb the girl to the point that she prefers not to take drugs. As for any use of antiepileptic medication, 16 of our patients were receiving treatment, which involved valproic acid in the majority of cases (6), levetiracetam (3), carbamazepine (2), oxcarbazepine (2), ethosuximide (1), topiramate (1), and lamotrigine (1).

### Measures

#### The Rorschach Test

The Rorschach test was administered, scored, and interpreted using Exner’s CS (42–45). This method was chosen for its good psychometric properties in terms of standardization, reliability, and validity, supported by several studies (37), although some drawbacks have to be cited, and first of all the time-consuming procedure and training necessary to submit and score this complex projective test. The last studies available (37, 46) regarding the validity of the Rorschach test scored and interpreted using the CS showed that *r* was 32 across 523 hypothesized relationships and 29 across 73 samples, whereas, correspondingly, the global validity of the MMPI was *r* = 0.32 across 533 hypothesized relationships and *r* = 0.29 across 85 samples.

The indexes obtained from the scoring are grouped into eight clusters synthesized in the structural summary: stress management and control, affective characteristics, perception of the self-image and interpersonal relationships, information processing, cognitive mediation, and ideation. Moreover special psychopathological indices are calculated form the scores, such as suicide constellation, perceptual thinking, depression, coping deficit, and hypervigilance index.

The affective features cluster, including for example the form: color ratio, the constriction ratio, and the presence of pure color, provides information about the way people experience emotions. These indexes differentiate people having adequate capacity to experience and express emotions from individuals inclined to process affects and emotional activation in an excessively intense or compulsory, disfiguring way, which implies consequent adjustment difficulties. The cluster regarding situational stress and control provides information on the individual psychological resources, ability to handle stress, and ability to confront consistently and effectively with life events. It includes, among others, the coping style (introversive versus extratensive), the human and inanimate movement, the difference score, and the sum of shading. The cluster of interpersonal perception helps in identifying whether a person is capable of sustaining a reasonable level of comfort in relationships and interpersonal interest, or *vice versa* if she/he is inclined to be disinterested, detached, or uncomfortable in social situations. Indexes, such as cooperative movement, poor human representation and personal responses can help to recognize if the individual is able to establish intimate and secure interactions or keeps distance and avoids proximity in order not to be hurt and if the person can establish a balance between collaboration-accountability and competitiveness-assertiveness, or has the tendency to become overly submissive/dominant in interpersonal relationships. Moreover the indexes pertaining to this cluster can discriminate between individuals who may accurately and empathically perceive other people’s acts in social situations, and those who may instead be inclined to misread the motivations of others and misunderstand the implications of interpersonal events. The cluster of self-perception provides information on how people see themselves, especially with respect to self-esteem, self-awareness, and self-image. These characteristics can be measured by indexes, such as morbid and reflections responses and the egocentricity index. The last clusters regard information processing, cognitive mediation, and ideational functioning, and measures how people focus attention on life events, perceive the environment and integrate perceptions. The styles of information processing are measured for example trough the synthesized or developmental quality vague (DQv) responses, perseverations, and organizational frequency while cognitive mediation can be individuated by popular, conventional, appropriate form, or *vice versa* distorted forms. This helps to evaluate if the individual perception of events and people is similar to the most frequently reported. Critical special scores, such as the weighted sum of the first six special scores, signal a potential disturbance of thinking processes, which are measured by the ideational functioning cluster. A successful adaptation indeed is promoted by an attitude of openness to new experiences, along with the ability to efficiently organize impressions and to perceive experiences in a realistic way. A logical, coherent, constructive, but also flexible and not too much conventional style of thinking can help to adaptively build and organize experiences and impressions about life events.

The emerging comprehensive picture of the personality functioning can be interpreted within a psychometric and psychoanalytic framework (43). Moreover the so-called “access keys” (43), identifying the more vulnerable characteristics, help in identifying disturbed areas in emotion regulation and thought processes, which may also entail a psychopathological disorders.

In this study, the computerized ROR-SCAN program (copyright 1988–2014 by Philip F. Caracena) was used for the interpretation in order to provide a structural overview and an interpretative report for each protocol. The variables analyzed were selected on the basis of the most recent meta-analysis concerning “The Validity of Individual Rorschach Variables: systematic Reviews and Meta-Analyzes of the Comprehensive System” (37). However, indexes were deliberately not too rigorously selected due to the absence of previous studies, in order to provide an exploratory analysis of these first data.

#### CBCL and YSR 11–18 (38)

The questionnaires, available in the proxy-report version for parents (CBCL) and in the self-reported one for adolescents (YSR 11–18), provide a profile of the adolescent skills and problem behaviors. The latter part consists of syndromic scales, scales of internalizing, externalizing, and total problems and scales oriented to the diagnostic categories of DSM IV. In particular, eight syndromes can be evaluated: social complaints, anxiety/depression, social problems, thought problems, attention problems, delinquent behavior, and aggressive behavior. Despite the limitations connected to the self-reported evaluations, the questionnaires allow, therefore, a preliminary clinical evaluation of the symptoms according to the DSM classification, which can be then confirmed by a diagnostic interview. The CBCL and YSR 11–18 are one of the most used measures of child and adolescent psychopathology. This multi-axial empirically based set of measures has been normed on an ample sample and was also standardized for the Italian population (47).

### Analyses

Descriptive statistics were used to describe the socio-demographic and clinical variables of the participants. Concerning the self-reported and proxy-reported questionnaires, after evaluating the distribution of data with the Kolmogorov–Smirnov’s test, the between group differences were tested using the Kruskal–Wallis test; differences were considered significant with a level of probability of *p* < 0.05.

A log-linear model approach for categorical variables was applied in order to compare the Rorschach scorings of patients with migraine with patients with epilepsy and with control adolescents (48). Models for categorical data are consistently used in psychological research [e.g., Ref. (49–52)]. The log-linear analysis in fact allows to estimate parameters that define the significant interactions between indicators measured by the Rorschach test and groups of patients. Data were cross classified by means of contingency bivariate tables, where each cell of the tables represented the interaction of each category of the first variable (the group) with each category of the second variable (Rorschach indexes): estimated parameters measure the strength of the association between variables for each cell of the tables. Interaction parameters and SEs were estimated to calculate a standard score for each interaction. These standardized parameters are presented in Table 2, with statistically significant values evidenced by the corresponding probability errors (*p < 0.05, **p < 0.01).

## Results

Concerning the proxy-reported CBCL scale, administered to mothers, higher scores for internalizing symptoms and in particular anxiety symptoms and somatic complaints were found in patients with migraine compared to patients with epilepsy group and controls. In these scales, patients with migraine more frequently had scores in the borderline or clinical range. As regards the self-reported YSR, higher scores for somatic complaints were found in patients with migraine compared to the adolescents belonging to the control groups; self-reported lower scores in anxiety symptoms were also found, as shown in Table 1.

**Table 1:** Psychopathological symptoms measured using the proxy-report questionnaire CBCL and the self-report questionnaire YSR in migraine, epilepsy, and control groups.

Scale	Migraine (median)	Epilepsy (median)	Control (median)	Kruskal–Wallis test (*p*)
CBCLInternalizing problems	59	50	50	7.41*
CBCL anxiety disorders	60	52	55	6.12*
CBCL somatic problems	66	52	54	23.65***
YSR 11–18 anxiety disorders	51	51	55	6.53*
YSR 11–18 somatic problems	63	51	53	17.53***

*CBCL, child behavior checklist; YSR 11–18, youth self-report*.

*p < 0.05.

***p < 0.001.

In Table 2, results concerning the Rorschach indicators are shown. Significant differences were found concerning the psychopathological index coping deficit index (CDI), affective features [see in particular form-color ratio (FC: CF + C)], situational stress and control [see difference score (D Score)], interpersonal perception [see personal (PER) responses], self-perception [see morbid (MOR) and form dimension (FD) responses], information processing [see perseverations and DQv responses], and ideational functioning [see weighted sum of the first six special scores (WSum6)].

**Table 2:** Log-linear standardized estimated parameters to compare the migraine, epilepsy, and control groups in relation to Rorschach indexes.

Clusters	Indexes	Scores	Migraine	Epilepsy	Control
Psychopathological indices	CDI	0	−1.68*	1.62	0.01
		1	1.68*	−1.62	−0.01

Affective features	CF + C > FC	0	−2.68**	1.18	0.64
		1	2.68**	−1.18	−0.64

Situational stress and control	D < 0	0	0.84	−2.53**	1.67*
		1	−0.84	2.53**	−1.67*

Interpersonal perception	PER	0	−2.44**	−0.06	1.50
		1	2.44**	0.06	−1.50

Self-perception	MOR	0	−2.67**	−0.39	2.04*
		1	2.67**	0.39	−2.04*

	FD	0	−2.57**	−0.96	2.93**
		1	0.47	1.77*	−1.46
		>1	1.91*	−0.77	−0.70

Information processing	PSV	0	−1.73*	0.28	0.97
		1	1.73*	−0.28	−0.97
	DQv	0	−1.80*	0.90	0.37
		1	1.80*	−0.90	−0.37

Ideational functioning	WSum6	0	−0.67	−0.97	1.26
		1	−1.05	0.97	0.29
		>1	2.067*	−0.42	−1.17

*CDI, coping deficit index; CF + C > FC, form-color ratio; D < 0, difference score (D Score) lower than 0; PER, personal; MOR, morbid; FD, form dimension; PSV, perseverations; DQv, developmental quality vague responses; WSum6, weighted sum of the first six special scores*.

*p < 0.05.

**p <0.01.

Among the psychopathological indices, the CDI was higher in patients with migraine. Concerning the affective features adolescent patients with migraine were found to present a higher sum CF + C (CF + C > FC) compared to patients with epilepsy and control adolescents. In fact as much as five pure color (C) responses were present in patients with migraine, three analogous responses were found in patients with epilepsy, while no one appeared in control adolescents. Moreover in patients with migraine, five M-responses and three M none responses were present whereas in the control adolescents such responses were completely absent. Concerning the situational stress and control, significant interactions were found between the three groups of adolescents and the difference score (D Score). Patients with epilepsy in fact more often are included in the category D < 0 (high situational stress, low control).

As regards interpersonal perception, adolescents with migraine more often presented a higher level personalized responses (PER), while the self-perception was characterized by an increased level of morbid (MOR) responses and FD responses; on the contrary the adolescents of the control group more frequently did not present with these kinds of answers. Moreover, patients with migraine were the only adolescents showing a more marked presence of perseverations and DQv responses, in comparison to patients with epilepsy and adolescents of the control group. Along with these significant results concerning the information processing, the ideational functioning appeared to be compromised in patients with migraine, who showed a higher weighted sum of the first six special scores (WSum6).

## Discussion

In the parent reported questionnaires, the migraine group obtained higher scores in internalizing problems and in particular in anxious and somatic symptoms. These findings are in line with the literature data indicating higher levels of internalizing, anxious, and somatic problems in children and adolescents with migraine compared to healthy controls (17, 23). There are, however, no statistically significant differences between the epilepsy group and the healthy control group, differently from Pinquart and Shen’s meta-analysis (53), where high levels of internalizing problems were found in all types of chronic illnesses analyzed, including headache and epilepsy (54, 55). Nevertheless, in line with the meta-analysis (53) on anxiety symptoms in patients with chronic illness, patients with migraine and epilepsy reported less anxiety symptoms in self-reports, compared to their parents’ evaluations. Different authors suggested that such a result may indicate a denial or underestimation of anxiety in children with chronic diseases, who may resort to an adaptive repressive style of managing negative emotions (53, 56, 57).

Since patients, differently from their parents, described themselves as distant from negative emotions, except for pain and somatic complaints, a merely self-descriptive approach, which implies good self-awareness and insight, would appear inadequate to describe patients’ affectivity and emotion management. Nevertheless, using a multi-method approach, including also a projective test that goes beyond defenses, has helped highlighting all the intensity and complexity of these adolescents’ emotional world.

Considering the projective test, the most notable differences between patients with migraine, patients with epilepsy and control adolescents were indeed related to the emotion management style. Among the variables of the affective cluster, significant differences emerged in relation to the form-color ratio (FC:CF + C), which is considered a variable with good support in the most recent meta-analysis on the topic (37). It allows to understand how the subject modulates affects, emotions, and their manifestations: in particular, the form-color (FC) responses represent more controlled emotional experiences, the color form (CF) are related to more spontaneous and freer emotional expressions, while the pure color (C) indicates a total absence of any form of affective modulation. Therefore, according to Exner standardization (42), the predicted proportion expected in adults as well as in adolescents is FC > CF + C, which corresponds to a good degree of affective control and modulation capacity. Individuals with a sufficient affective modulation are able to engage in situations with an affective value and to exchange feelings with others; they tend to recognize the feelings expressed by others and allow their feelings to emerge and be recognized by those around them.

Differently from what happen in this ideal adaptive situation, adolescents with migraine were found to have a greater level of less controlled emotional experiences (CF + C > FC) compared to patients with epilepsy and to healthy controls. A high value on the right side of the relationship may indicate that emotional behaviors are characterized by impulsivity and intensity. The CF responses highlight individuals whose emotions direct behaviors, while pure color point to even less adaptive responses, such as impulsivity. Crumpton (58) found that color tables tend to induce less pleasant and more aggressive reactions than achromatic tables: these affective patterns, revealed by the colored tables of the Rorschach test, seem to correspond to the ability in adapting to reality, which appeared more compromised in the migraine group where the color responses are less modulated and mature than expected for age (42).

Parallel to this finding, also the CDI, which was higher in patients with migraine, indicated an immature personality organization producing vulnerability in managing the demands of everyday life. These difficulties appear marked in the interpersonal sphere and may contribute to problems in control capacity. Since patients with migraine have difficulties in managing feelings and expressing them, emotions are experienced with much higher intensity. Such a turbulent and raw emotional experience appears in line with the construct of alexithymia, an affective-cognitive style characterized by a poor ability of experiencing and expressing emotions (59), which is often recalled in studies that underline the role of the psychosomatic role in chronic disorders (60) This style of emotion management was in fact shown to lead to an increased risk of developing psychopathological as well as somatic symptoms (61, 62), is kept with some pioneering studies conducted on children and adolescents with headache and migraine (29, 30, 33, 63).

Concerning the ability to control and manage situational stress, differences among the three groups were found in the difference score, which is considered a variable with good support (37) providing a general evaluation of the dynamics between the subject’s total (ideative and affective) resources and the levels of stress and discomfort (ideative or affective or stable) experienced. This index allows to detect situational stress, to measure its intensity, and the risk of resorting to impulsive behaviors. When D < 0 the patient experiences a state of overload: the stress is higher than he can deal with efficiently. In this situation, the individual has a poor control over his decisions and behaviors are poorly elaborated and scarcely modulated; a vulnerability to disorganization situations is, therefore, present when the subject is confronted with complex or ambiguous stimuli. Our findings showed that patients with epilepsy are most likely to present these characteristics, probably due to the chronic disease, which implies a greater amount of stress experienced (55). Although results were not significant in this sense, also patients with migraine could be hypothesized to experience more stress compared to controls: they appear indeed more prone to impulsive acting-out. In fact negative and undetermined Human Movement Responses (M-, M none) in addition to Pure Color responses, which are completely absent in the control adolescents, were observed only in patients with migraine, indicating a lack of control on their own experiences and emotional reactions. This finding once more highlights migraineurs’ more marked difficulties in using their cognitive resources to modulate affections, without resorting to impulsive behavior or somatic symptoms in order to cope with emotional tensions (64).

These patients’ characteristics may be strictly related to a particular perception of interpersonal relationships: adolescents with migraine in fact more often resort to personalized responses (PER) during the Rorschach test, referring to their personal experiences. This reflects the need for these individuals to defend their own points of view and affirmations, in order to protect themselves from the clinician and from the situation of the test, and consequently also in everyday situations, from anyone considered to be judging or threatening them. This mechanism derives from the need to reduce insecurities by showing almost imposing personal certainties with a sort of defensive intellectual authoritarianism that could deeply disturb the relations with others.

On the other hand, the patients’ insecurities may reflect a perception of a negative and deficitous self, as shown by the increased level of morbid responses [MOR—variable with good support, (37)] in patients with migraine compared with the adolescents belonging to the control group. In this kind of responses in fact an object is identified as dead, destroyed, damaged, deteriorated, ruined, injured, or broken, which may represent a disqualifying self-perception both at the affective and the ideative level. This can be linked with the tendency to obsessive ruminations, showed by the increased FD responses [variable with little support, (37)] which implies an impression of spatiality/depth/dimensionality determined by formal characteristics, and thus represents the ability to look in perspective. Such an introspective tendency can lead to personal growth (adaptive outcome) or, when it is excessive, to a sterile self-critique (maladaptive outcome).

Concerning the quality of the information processing, the more marked presence of perseverations suggests that adolescents with migraine can be characterized by a sort of mental stiffness that prevents them from modifying their own thoughts and opinions with adequate flexibility. In these patients, the significant presence of perseveration along with the high values of FD responses might reflect a negative image of self and a greater fear of others’ judgment, which may be compensated by showing lots of personal certainties and an authoritarian style (more personalized responses). Moreover, a greater number of responses with vague quality suggests a less sophisticated and immature processing quality in patients with migraine. The DQv index indeed denotes an approach to both internal and external reality rather vague and poorly defined.

Along with a more vague and immature approach to reality, also negative ideative characteristics emerged, which include a tendency toward less clear thinking and a more immature and less sophisticated conceptualization. Patients with migraine presented more significant pathological aspects of thinking processes and cognitive impairments compared to patients with epilepsy and controls, as shown by the increased level of the weighted sum of the first six special scores (WSum6), which is considered an index with excellent support in Mihura’s meta-analysis (37). It is, therefore, worth noticing that the patients with epilepsy do not show significant differences in this specific aspect and in most of the Rorschach scores studied (excluding the high level of situational stress) compared to normal controls, although some patients are subjected to preventive drug therapy that, as known, may have some side effects, including behavioral problems and somnolence (65). The rate of adverse drug reactions, however, is demonstrated to be significantly lower in children and adolescents receiving monotherapy (65), like the patients included in this study. The single special score appeared not enough to indicate an overt disturbance of thinking processes in any patient; however, all the patients with migraine, differently from adolescents of the other two groups, gave at least some responses of this type. It is thus possible to hypothesize a greater fragility of thinking processes in these patients compared to epileptic patients and healthy controls. Although usually these patients can be considered closer to the normality than to a thought disorder, literature has shown that psychological parameters of high sensitivity may reveal important peculiarities in their functioning (66). These considerations suggest the need for further research, using also projective standardized test, in order to better understand the patients’ emotional and ideative characteristics.

Projective tests, such as the Rorschach test, have indeed the advantages of going beyond the individual defense mechanisms (such as tendency to negation) and being very sensitive also to the smallest variations in psychic functioning and personality characteristics, including those which represent only physiological variants of normality, such as character styles and personality traits. Despite these indubitable advantages, the administration of such complex and sophisticated investigations requires more training and time to the clinician compared to administration of self-reported questionnaires (such as CBCL and YSR 11–18), which is undoubtedly an important disadvantage for a cost-effective feasible research. Moreover, these procedures can be more challenging and emotionally tiring even for the patient. On the other hand, projective techniques allow a deeper understanding of the individual, thus facilitating both the diagnostic and the therapeutic procedures. As to this last point, the complete profile and the wide set of information made available by the Rorschach test may be an important support to a psychological care plan, which can act in parallel with pharmacological therapy, only aiming at pain control. Literature and clinical experience with child and adolescent migraine converge to suggest that, as opposed to an aprioristic assessment of the painful symptom, detecting also dysfunctional psychological aspects through the interview with the patient and the parents represents an important turning point for improving the treatment possibilities and the quality of life of the patients themselves (67–69).

This study has some limitations. The samples are relatively small; this prevents both the possibility to draw definitive generalizable conclusions, and the possibility to reach significant results in a regression analysis intended to test the inter-relations between the different variables included in the clusters described in the manual of the CS (34). Moreover, the group of patients with epilepsy was defined by broad criteria, so that a more detailed statistical analysis in these patients was unsuccessful as well. Subsequent studies will be necessary to deal with these specific aspects, which could not be explored in this study, given the dimension of the sample. Confirmatory studies are, therefore, needed in larger samples of patients with migraine and epilepsy in order to confirm the present preliminary results.

Despite these limitations, the multi-method approach adopted in this study can give interesting indications for future more extensive research, since it allowed identifying potential personality difficulties within the group of adolescent with migraine, both in the cognitive and affective sphere. These difficulties seemed to be specific of patients with migraine compared to other adolescents with a chronic disease such as epilepsy (although some similarities were found between the two groups, concerning for example the high level of stress and the problems in coping whit it as a consequence of chronic suffering). In the analysis of the areas of affectivity, self-perception, interpersonal relationships, ideation, and stress control, a number of different limitations have been highlighted in patients with migraine, in particular regarding the difficulty in modulating the intensity of affects and emotions, which coexist with less conventional thinking processes. Along with appropriate areas of functioning, such limitations of psychological functioning suggest the characterization of the psychopathological comorbidities of migraine as prevalently psychological disorders rather than psychiatric ones. In line with previous studies (33), the results of this research may, therefore, support the hypothesis of an important contribution of emotional factors in the determinism and course of migraine. If migraine can be in part understood as a psychosomatic illness (70) nevertheless the findings of this study did not point to a particular energy and intensity of painful or negative feelings in these patients, but rather to a deficit or a transient psychic difficulty in the mental ability to tolerate and process such feelings and the related thoughts (28, 33). Subsequent studies, which would include an extension of the samples and possibly a longitudinal follow-up, might lead to a better understanding of personality and psychopathological aspects in adolescent patients with migraine and potentially reveal a prognostic value of the Rorschach test in respect to the adolescents’ outcome and quality of life.

## Ethics Statement

Patients and parents have given their written consent to the participation in the study in accordance with the national and institutional code of good ethical practice and with the Declaration of Helsinki. This study was conducted within the context of a wider research project, approved by the Ethical Committee of National Neurological Institute IRCCS C. Mondino on April 19, 2011.

## Author Contributions

LB, SM, and UB wrote the manuscript. UB, LB, SM, MC, AM, and DC contributed to study design, data discussion, and critical evaluation of the manuscript. AM and DC contributed to data collection and Rorschach scoring. SM and MC performed analyses and contributed to data interpretation. All the authors approved the final manuscript and agreed to be accountable for all the aspects of the study.

## Conflict of Interest Statement

The authors declare that the research was conducted in the absence of any commercial or financial relationships that could be construed as a potential conflict of interest. The reviewer PK and handling editor declared their shared affiliation.
